# Aqueous extract of *Saposhnikovia divaricata* root alleviates rheumatoid arthritis by acting on TNF-α and RAGE signaling pathways

**DOI:** 10.1016/j.bbrep.2025.102153

**Published:** 2025-07-12

**Authors:** Anjing Xu, Yuanyuan Wen, Bao Hou, Shijie Zhang, Tsedien Nhamdriel, Xiaoyue Ma, Liyuan Cui, Xuexue Zhu, Weiwei Cai, Liying Qiu, Haijian Sun

**Affiliations:** a MOE Medical Basic Research Innovation Center for Gut Microbiota and Chronic Diseases, School of Medicine, Jiangnan University, Wuxi, 214122, China; b Department of Basic Medicine, Wuxi School of Medicine, Jiangnan University, Wuxi, 214122, China; c Department of Basic Medicine, Tibet University of Medicine, 850000, Lhasa, China; d State Key Laboratory of Natural Medicines, China Pharmaceutical University, No. 24 Tongjia Lane, Nanjing, 210009, China

**Keywords:** *Saposhnikovia divaricata* (Turcz.) Schisch, TNF-α, RAGE, Rheumatoid arthritis, UPLC-Q-TOF-MS

## Abstract

Saposhnikovia divaricata (Turcz.) Schisch (SADS) is classified as a special-grade traditional Chinese medicine in Shennong's Materia Medical due to its immune-protective effects, including dispelling cold, relieving edema and pain, and its potential in treating rheumatoid arthritis (RA). Over 130 traditional Chinese medicine formulations containing SADS are used for RA treatment. However, the active ingredients and serum metabolites of SADS remain underexplored, and its precise mechanism of action in RA is not fully understood. Therefore, the study aims to explore the active ingredients and serum metabolites of SADS by UPLC-Q-TOF-MS and investigate its therapeutic mechanisms in the context of RA. A total of 5536 compounds were identified in SADS, and 19 active components were finally selected. In serum metabolites following SADS administration, 4945 compounds were identified, of which 17 showed anti-inflammatory activity. Network pharmacology analysis showed that SADS may play a role in the treatment of RA through the TNF and Receptor for Advanced Glycation End-products (RAGE) signaling pathway. SADS alleviated RA symptoms in IL-1RA deficient RA mice. In cellular models, SADS inhibited the abnormal proliferation of fibroblast-like synoviocytes through regulating the TNF-α and RAGE pathways. In addition, SADS promoted the polarization of M2 macrophages but inhibited the polarization of M1 macrophages. SADS alleviated the progression of experimental arthritis in a RA mouse model by modulating the TNF-α and RAGE signaling pathways, supporting its potential as a therapeutic agent for RA.

## Highlights

• Among the serum metabolites after SADS administration, 17 compounds with anti-inflammatory activity were identified.
• SADS active ingredients and serum metabolites interfere with RA through TNF-α and RAGE signaling pathways.
• SDS alleviated the progression of RA by regulating TNF-α and RAGE signaling pathways.

## 1 Introduction

RA is a chronic autoimmune inflammatory disease characterized by synovial hyperplasia, joint deformity and inflammatory infiltration [1]. The global incidence of RA is approximately 1 %, with women over 40 years old being particular at high risk [2]. The pathogenesis of RA is very complex, both genetic factors (DNA methylation and histone acetylation) and environmental factors (such as smoking, dust, and obesity) are closely related to RA development [3,4].

Currently, Disease-Modifying Anti-Rheumatic Drugs (DMARDs) are categorized into several types, including conventional synthetic DMARDs, such as methotrexate, biologic DMARDs, such as tumor necrosis factor-α (TNF-α) inhibitors and interleukin-6 (IL-6) inhibitors; and targeted synthetic DMARDs, such as Janus kinase (JAK) inhibitors. These agents have demonstrated significant efficacy in the treatment of rheumatoid arthritis (RA) [5]. In addition to DMARDs, glucocorticoids and non-steroidal anti-inflammatory drugs (NSAIDs) are commonly used as adjunct therapies to manage inflammation and symptoms in RA. Among glucocorticoids, low-dose oral prednisolone is the most frequently prescribed in clinical practice [6]. Other agents, such as triamcinolone, dexamethasone, and betamethasone, are also used in specific contexts [7]. While glucocorticoids are effective for short-term symptom control, long-term use, particularly at high doses, can lead to adverse effects such as osteoporosis and hypertension [8]. NSAIDs, including salicylic acids, arylpropionic acids, acetic acids, and pyrazolones, exert their anti-inflammatory and analgesic effects by inhibiting cyclooxygenase enzymes and subsequently reducing prostaglandin production [9,10]. However, their clinical utility may be limited by gastrointestinal, renal, and cardiovascular side effects [11]. Moreover, Traditional Chinese Medicine (TCM), with its holistic and multi-targeted approach, has shown promise as a complementary or alternative therapeutic strategy for RA management [12].

SADS, the dried roots of the Umbelliferae plant SADS, is a TCM widely used to treat diseases of nervous and immune systems [13]. In Asian countries, SADS had been widely used in TCM prescriptions and Chinese patent medicines for the treatment of RA [14].The Chinese Pharmacopoeia clearly recorded that SADS had the effect of treating rheumatoid arthritis [15]. Jiang et al. found that SADS had a significant therapeutic effect on collagen-induced arthritis (CIA)mice and that Prangenidin, the main component of SADS, could induce apoptosis of fibroblast-like synoviocytes through PI3K/AKT pathway [16]. Li et al. found that SADS could effectively alleviate RA symptoms and regulate metabolic disorders in CIA rats [13]. In “Shen nong *Materia Medica*”, SADS is listed as a special grade TCM because of its immuno-protective effect of dispelling cold, relieving pain caused by edema and anti-spasm [17,18].

The crude SADS extract contained a large number of chromones, coumarins, polyacetylenes, and acid esters and these compounds have significant anti-inflammatory, analgesic, and immunomodulatory activities [17]. Active substances in SADS extracts, such as prime-*O*-glucosylcimifugin, 4′-*O*-β-d-glucosyl-5-*O*-methylvisamminol, 5-*O*-methylvisamminol, and anomalin, have been reported to possess anti-inflammatory and analgesic effects [14,19,20]. However, the active ingredients and their mechanisms of action in the bloodstream remain unclear after SADS is absorbed. To address this gap, LC-MS technology was employed to identify the active components of SADS in serum, and network pharmacology was used to elucidate the action pathways of these components.

**Scheme 1:**
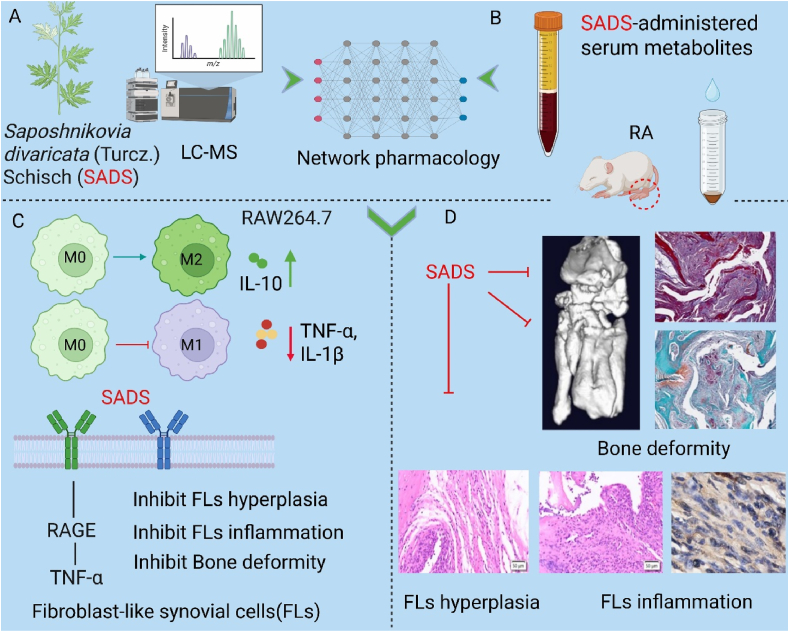
(A and B) Screening of active substances in SADS and SADS-administered serum metabolites and network pharmacology screening targets. (C) Exploring the molecular mechanism of SADS effect on RA. (D) SADS ameliorated RA symptoms in spontaneous IL-1RA^−/−^ mice.

In this study, we aimed to characterize the anti-inflammatory activity of SADS in serum by serum pharmacochemistry and network pharmacology approaches and to uncover the underlying mechanism of action of SADS in RA treatment. First, the therapeutic effect of SADS on RA was evaluated by using a spontaneous RA mouse model. The active components of SADS in serum were detected by LC-MS, followed by the enrichment analysis of the selected anti-inflammatory active ingredients using network pharmacology. The “ingredient-gene-pathway” network was then constructed. Finally, the network pharmacology results were validated at the cellular level (Scheme 1).

## 2 Materials and methods

### 2.1 Reagents and chemicals

*Saposhnikovia divaricata* (Turcz.) Schisch (No: 20240111) was purchased from Xunbaicao Wild Chinese Herbal Medicine Co., LTD (Chengdu China). RAW264.7 macrophages, TNF-α (BA0131) and IL-10 (A00021-3) primary antibodies were purchased from Boster Biological Technology Co.,LTD(Wuhan China). Fibroblast-like synoviocytes was purchased from Wuhan Punosay Life Technology Co., LTD(Wuhan China). IL-1β (16806-1-ap) and advanced glycation end products (RAGE) (66833-1-Ig) primary antibodies were obtained from Wuhan Sanying Biotechnology Co., LTD (Wuhan China). CD206 (A02285-2) and CD86 (BM4121) primary antibodies were purchased from Boster Biological Technology Co.,LTD (Wuhan China). TNF-α (MM-47831M2) and IL-6 (MM-1011M2) ELISA Kits were purchased from Jiangsu Meimian industrial Co., LTD (Jiangsu, China). Saffron-O and Fast Green Kit (G1371) was purchased from Beijing Solarbio Technology Co., LTD (Beijing China). d-Ribose (HY–W018772) was purchased from Medchemexpress Co., LTD (New Jersey, USA).

### 2.2 Cell culture

The RAW264.7 macrophages were purchased from Procell system (CL-0190, Wuhan, China) and were cultured in DMEM high-glucose medium containing 10 % fetal bovine serum. Human fibroblast-like synoviocytes were purchased from Procell system (CP–H241, Wuhan, China) and were cultured in DMEM containing 10 % FBS and 1 % triple-antibiotic solution (penicillin-streptomycin-amphotericin B). The conditions in the cell incubator were set to: temperature 37 °C, 5 % CO_2_. When the cell proliferation reached 80 %–90 % confluence, the next passage and plating steps were carried out. First, the cell culture medium in the culture dish was discarded, and the cells were washed 3 times with an appropriate amount of pre-warmed phosphate-buffered saline (PBS) solution. Then, the cells attached to the bottom of the dish were detached by pipetting, mixed thoroughly, and collected into a cell suspension. The cell suspension was appropriately diluted, and the cell count was performed using a hemocytometer under a microscope. The required number of cells were plated according to the specific experimental requirements.

### 2.3 Transwell experiment

After 12 h of starvation treatment of fibroblast-like synoviocytes, the cells were digested with trypsin and resuspended in high-glucose medium without serum. The cells were seeded at a density of 50,000 cells/well in the chambers. The lower chamber was filled with high-glucose medium containing serum and drugs, and the cells were cultured for 24 h in a 37 °C, 5 % CO_2_ incubator. After fixation for 20 min, the cells were stained with crystal violet for 15 min and photographed.

### 2.4 Preparation of aqueous extracts of SADS

SADS (120 g) was weighed accurately and mixed with 1200 mL of distilled water, then heated in a water bath at 65 °C for 24 h. After filtration through 16 layers of gauze, the solvent was evaporated using a rotary evaporator to yield a concentrated paste. The paste was then frozen at −80 °C for 24 h and lyophilized to obtain the dry powder.

### 2.5 Drug target and disease target screening

UPLC-Q-TOF-MS was performed to identify the phytochemical components in SADS water extract. Untargeted metabolomics was used to identify compounds in SADS aqueous extracts, and active substances were sequentially screened based on the criteria of matching scores greater than 50 and mass errors less than 1.

### 2.6 Drug target and disease target screening

To explore the potential pharmacological mechanisms of SADS in treating RA, we conducted a systematic in silico analysis using publicly available databases. The chemical components and associated targets of SADS were obtained from the Traditional Chinese Medicine Systems Pharmacology (TCMSP) database (http://tcmspw.com/tcmsp.php). Screening criteria were set as oral bioavailability (OB) ≥ 30 % and drug-likeness (DL) ≥ 0.18 to identify bioactive ingredients. Canonical SMILES of these active compounds were then retrieved from PubChem (https://pubchem.ncbi.nlm.nih.gov/) and imported into the SwissTargetPrediction database (http://www.swisstargetprediction.ch/) to predict potential human protein targets. To identify disease-related targets, “rheumatoid arthritis” was used as a keyword to search the GeneCards database (https://www.genecards.org/). All retrieved targets were standardized and deduplicated before further analysis. It is important to note that this step involves computational predictions and does not reflect direct experimental validation.

### 2.7 Venn diagram construction

The predicted targets of SADS and the known targets of RA were uploaded to the Microbio-Xin Online Bioinformatics Analysis and Visualization Cloud Platform (https://www.bioinformatics.com.cn/) to generate a Venn diagram. The overlapping genes between drug-related and disease-related targets were identified as potential therapeutic targets of SADS in RA. These intersection targets served as the basis for subsequent network construction and pathway enrichment analysis.

### 2.8 Construction and analysis of protein-protein interaction (PPI) networks

The intersection targets obtained were imported into the STRING database (https://cn.string-db.org/). After selecting “multiple Proteins”, the restricted species was selected “Homo sapiens”, the confidence was set to medium (0.4), and the nodes with interrupted network were hidden. The PPI network map of intersection genes was obtained and saved in TSV and PNG format. Import the file in “TSV” format into Cytoscape3.10.2 software. Based on the Centiscape2.2 function, the targets with Degree, Closeness and Betweenness greater than the average were screened out, and the redundant targets were deleted. Based on the “Network Analyzer” function, the Degree value is obtained. According to the Degree value, the obtained targets were arranged in descending order to obtain the related information of the core targets. Export as image.

### 2.9 GO and KEGG pathway enrichment analysis

GO and KEGG pathway enrichment analyses were performed using the European Yi Yun platform (https://cloud.oebiotech.cn/). The input included the core targets, and the top 20 GO functions and KEGG pathways were analyzed.

### 2.10 Enzyme linked immunosorbent assay (ELISA) for serum cytokine levels

The serum levels of TNF-α (EK0527, Boster, Wuhan, China) and IL-10 (EK0417, Boster, Wuhan, China) in control and IL1RA-deficient mice were measured by commercial ELISA kits following the manufacturer's protocols. In brief, the pre-sealed 96-well plate was equilibrated to room temperature. Standard and serum samples (100 μL each) were added to the wells and incubated at 37 °C for 1.5 h. After discarding the reaction solution, 100 μL of biotin-labeled antibody (1:100 dilution) was added, and the plate was incubated at 37 °C for 1 h. Washing was performed three times with wash buffer for 10 minh. Then, 100 μL of ABC solution (1:100 dilution) was added and incubated at 37 °C for 30 min. The plate was washed five times with wash buffer, with each soak lasting 1.5 min. The plate was incubated in the dark at 37 °C for 20 min with 90 μL TMB substrate. After adding 100 μL of termination solution, the OD value at 450 nm was measured, and the concentration of the measured substance was calculated using ELISA software.

### 2.11 Real-time PCR

Total RNA was extracted by Vazyme RNA extraction kit and cDNA was synthesized by Yeasen reverse transcription reagent kit. Real-time PCR was performed with the Hieff® qPCR SYBR Green Master Mix(Low Rox Plus) kit (yeasen). The reaction conditions were as follows: denaturation at 95 °C for 5min; A total of 40 cycles of 95 °C for 10 s for melting, 60 °C for 30 s for annealing and extension were performed. The CT values of each sample were homogenized by the reference gene β-actin.

### 2.12 Animal experiments

The interleukin-1 receptor antagonist (IL-1Ra) is a natural inhibitor of interleukin-1 (IL-1), a key proinflammatory cytokine involved in various inflammatory processes [21]. IL-1Ra prevents IL-1 from binding to its receptor, thereby suppressing the production of downstream proinflammatory mediators such as IL-1β, IL-6, and TNF-α [22]. It has been demonstrated that mice lacking the IL-1Ra gene under Balb/c background spontaneously develop autoimmune and joint-specific inflammation resembling RA [[23], [24], [25], [26]]. IL-1 receptor antagonist-Deficient (IL1RA^−/−^) mice on a BALB/c background were constructed and provided by GemPharmatech (Strain No. T058785, Nanjing, China). Mice were maintained at constant temperature and humidity room with a standard 12h/12h light/dark cycle, an average temperature of 21–22 °C, and 50–70 % relative humidity. All the mice were fed adaptively for one week before being used in experimentation, and all experimental animals were provided with a standard diet. Mice had free access to standard chow and water. Thirty 4-week-old male IL1RA^−/−^ mice were randomly divided into five groups: control group (Ctrl), Model group (Model), SADS water extracts low-dose group (SADS-L), SADS water extracts high-dose group (SADS-H) and methotrexate group (MTX). Group SADS-L was given 63 mg kg^−1^ by gavage, group SADS-H was given 126 mg kg^−1^ by gavage and group MTX was administered by intraperitoneal injection at a dose of 2 mg/kg every 3 days [27]. Treatments started when RA symptoms appeared (week 4) and continued until week 9. From day 27 on, the mice were weighed every 3 days, and the arthritis index of the left and right feet was measured every 5 days. The therapeutic effect of SADS was evaluated at the end of the experiment. All animal experiments were approved by the Experimental Animal Ethics Committee of Jiangnan University (Ethics Batch No: JN.No20240530 t030 0915). The degree of foot swelling was clinically scored every 4 days after intragastric administration. After 5 weeks of drug administration, the foot status of mice was photographed and recorded. Clinical score: no erythema and swelling on the foot, 0; Erythema or mild swelling of the ankle joint, 1 point; Erythema and mild swelling from foot to ankle, 2 points; Erythema and moderate swelling of ankle joint to metatarsal joint, 3 points; Erythema and severe swelling of ankles, toes, or limbs, 4 points [28].

### 2.13 Micro-CT scan

Micro-CT (quantum GXII, PE, Germany) was used to detect foot deformities and ankle bone volume fraction (BV/TV), among others, in mice. The scanning conditions were set as follows: scanning voltage: 90kv, scanning current: 80 μA, scanning field of view: 16 mm, scanning time: 4min, scanning mode: high resolution, filter: Cu0.1, scanning layer thickness: 36 μm.

### 2.14 H&E staining

Mouse ankles were fixed in 4 % paraformaldehyde for 10 days followed by a 4-week decalcification process [29]. After decalcification, the ankle joint was embedded using paraffin and cut into 5 μm sections. Sections were deparaffinized prior to H&E staining, and tissue sections were then hydrated with gradient ethanol and stained with a hematoxylin-eosin staining kit. The pathological changes of the ankle joint were observed using a digital slice scanner.

### 2.15 Immunohistochemistry (IHC) staining

The ankle joint samples of the mice were analyzed by IHC staining. After paraffin sections were deparaffinized and rehydrated, the samples were antigen-repaired, membrane-broken, and blocked at room temperature. Subsequently, the samples were incubated with specific primary antibodies at a dilution of 1:200 overnight at 4 °C. The next day, tissue sections were rewarmed at 37 °C for 1h, washed with PBS buffer, and then stained with horseradish peroxidase (HRP) -labeled secondary antibodies for 30 min at room temperature and nucleated with hematoxylin for 30s. The slices were sealed after transparent treatment. Finally, the expression of relevant genes in the ankle joint was observed using a digital slide scanner.

### 2.16 Masson staining

Paraffin sections of the ankle joint were de-paraffinized, and the sections were sequentially placed in xylene, absolute ethanol, alcohol gradients, and finally rinsed with distilled water. Then, nuclei were stained with Weigert iron hematoxylin staining solution for 5–10 min. A further 5–10 min of staining was performed with Ponceau fuchsin staining solution. Finally, aniline blue staining was used for 3 min. Finally, it is dehydrated and transparent.The scores of bone destruction, cartilage and osteogenesis damage were 4 points, osteogenic damage was 3 points, total cartilage damage was 2 points, local cartilage damage was 1 point, cartilage and osteogenesis without damage was 0 point.

### 2.17 Statistical analysis

All data are expressed as the mean standard deviation (SD). Graph prism 10.1.2 software was used for statistical analysis. Independent sample *t*-test was used for comparison between the two groups, and one-way analysis of variance (ANOVA) and Dunnett test were used for multiple group comparison. Differences were considered significant at P < 0.05.

## 3 Results

### 3.1 UPLC-Q-TOF-MS identification the main active components in SADS

**Fig. 1:**
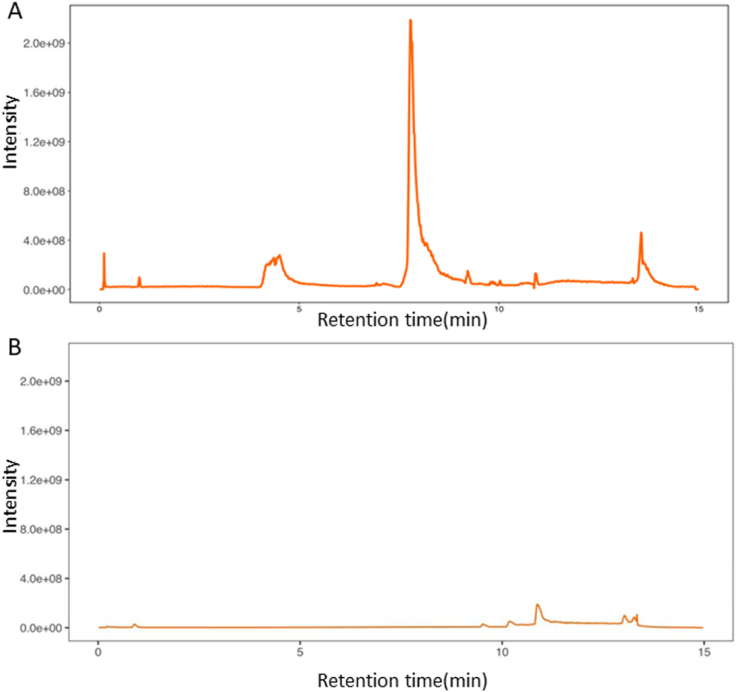
Total ion current chromatograms of SADS in positive (A) and negative (B) modes.

**Fig. 2:**
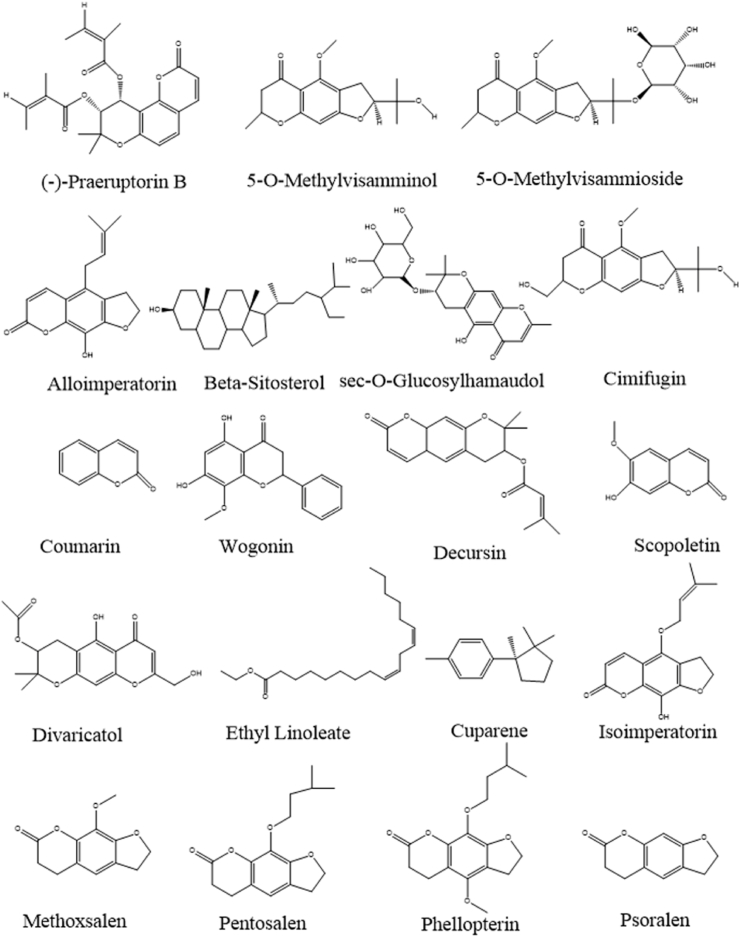
The structures of 19 compounds in SADS were identified by UPLC-Q-TOF-MS.

UPLC-Q-TOF-MS is widely used in Chinese medicine, pharmacy and clinical medicine [30]since it has high sensitivity and accuracy for the analysis and identification of TCM components and drug metabolism *in vivo* [31]. Therefore, in this study, UPLC-Q-TOF-MS was performed to identify the phytochemical components in the aqueous extract of SADS. The positive and negative ion chromatograms of the SADS water extract are shown in Fig. 1A and B. Using a non-targeted metabolomics approach, a total of 5536 compounds were identified in the extract. Among these, 643 compounds with matching scores greater than 50 were selected, and 19 compounds with mass errors less than 1 were further refined. These 19 compounds were primarily coumarin derivatives, including sec-*O*-glucosylhamaudol and scopoletin, which are known for their anti-inflammatory and analgesic effects [[32], [33], [34], [35]]. Additionally, beta-sitosterol [36], cimifugin [37], wogonin [38], and phellopterin [39] exhibited notable anti-inflammatory and immunomodulatory activities. The structural formulas of these compounds are presented in Fig. 2.

### 3.2 Target genes were predicted and related pathways were enriched by active ingredients of SADS

**Fig. 3:**
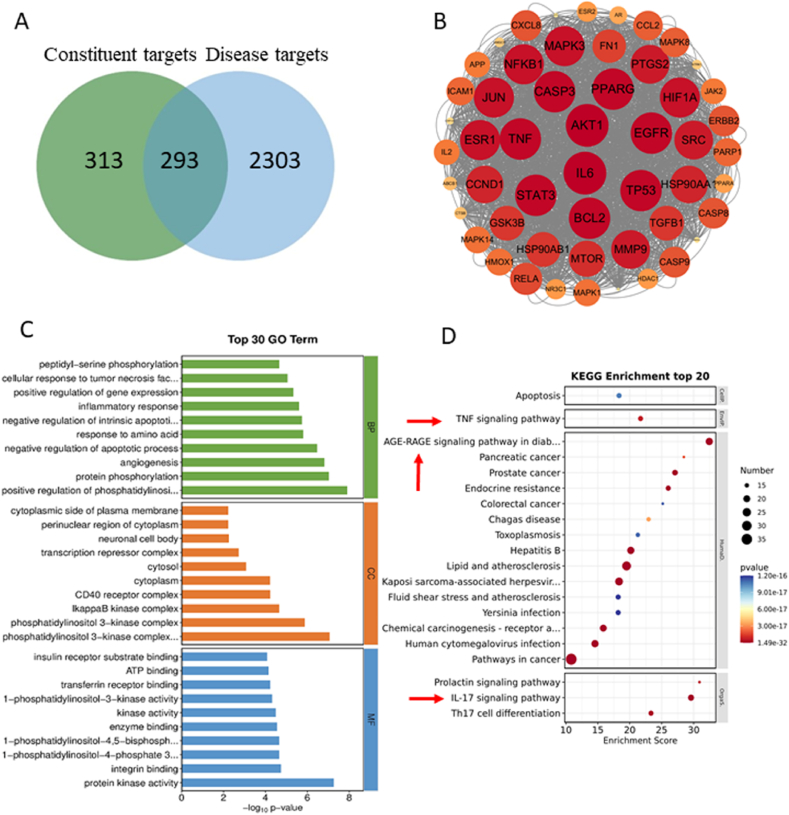
SADS active ingredients targets and RA disease targets, as well as GO and KEGG pathway enrichment analysis. (A) Venny diagram of intersection between SADS targets and RA targets. (B) PPI network diagram of the targets of SADS in the treatment of RA. (C)GO enrichment analysis. (D)KEGG enrichment analysis.

Through a public database, 19 compounds screened from SADS were predicted for drug targets, and 606 targets were screened. The keyword “RA” was then used to screen for disease-related targets, yielding 2596 targets. After intersecting the drug and disease targets, 293 potential therapeutic targets of SADS in RA were identified (Fig. 3A).To comprehensively analyze the potential mechanism of SADS in RA, 52 targets were selected based on the “Centiscape2.2″ function, using degree, closeness, and betweenness values greater than the average. The protein-protein interaction (PPI) network was then constructed using the STRING database (Fig. 3B). GO and KEGG pathway enrichment analyses were performed on these 52 targets. GO analysis revealed that these targets were involved in processes such as the inflammatory response, peptidyl-serine phosphorylation, and apoptotic processes. Cellular component (CC) analysis emphasized the role of the phosphatidylinositol 3-kinase complex and IκB kinase complex. Molecular function (MF) analysis highlighted protein kinase activity (Fig. 3C). The primary KEGG pathways targeted by SADS in RA included the TNF signaling pathway, RAGE signaling pathway, and IL-17 signaling pathway (Fig. 3D).

### 3.3 SADS alleviated inflammation in fibroblast-like synoviocytes and macrophages

**Fig. 4:**
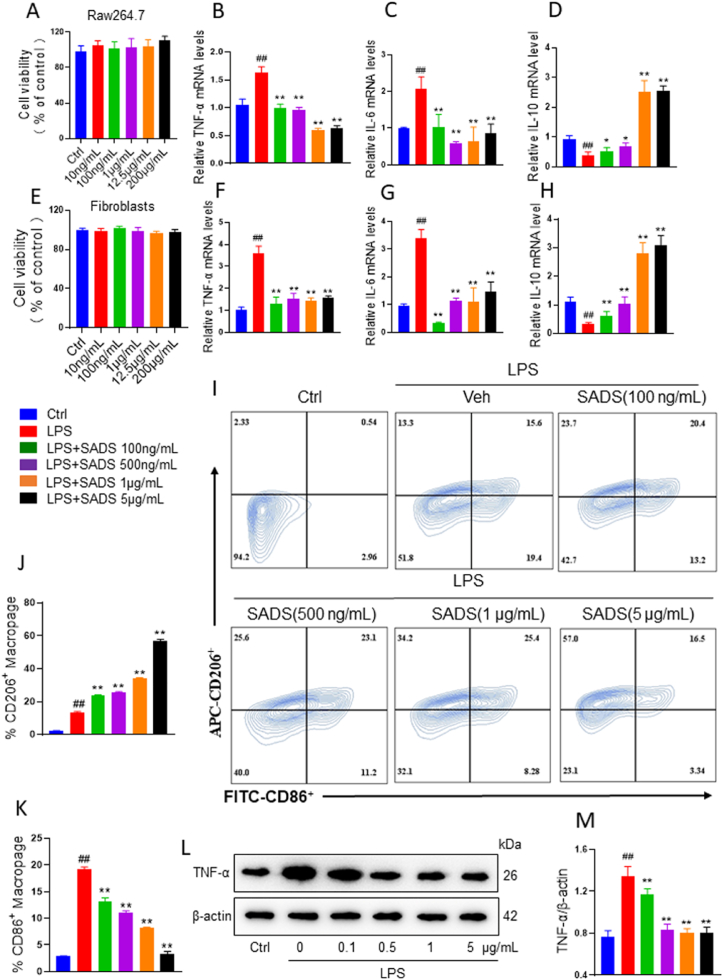
SADS inhibited Raw264.7 and fibroblast-like synoviocyte inflammation *in vitro*. (A) The effect of SADS on the viability of Raw264.7. (B–D) RT-PCR analysis of TNF-α, IL-6 and IL-10 in Raw264.7 treated with SADS.(E) The effect of SADS on the viability of fibroblast-like synoviocytes. (F–H) RT-PCR analysis of TNF-α, IL-6 and IL-10 in fibroblast-like synoviocytes treated with SADS.(I) The phenotype of Raw264.7 was analyzed by flow cytometry.(J and K) Statistics of the proportion of M2 and M1 macrophages. (L and M) TNF-α protein expression level detection. ##P < 0.01 versus Ctrl; ∗∗P < 0.01versus LPS. n = 6.

As shown in Fig. 4A and E, SADS had no significant effect on the cell viability of Raw264.7 and fibroblast-like synoviocytes within the concentration range of 10 ng/mL to 200 μg/mL. LPS treatment significantly upregulated the expression of TNF-α and IL-6 in both Raw264.7 and fibroblast-like synoviocytes. However, SADS notably reversed the LPS-induced increase in these cytokines (Fig. 4B and C and 4F-G). Further analysis revealed a significant reduction in the mRNA expression of IL-10 in the LPS group, while SADS treatment significantly elevated IL-10 expression (Fig. 4D and H). Flow cytometry analysis indicated that SADS induced M2 polarization and suppressed M1 polarization in Raw264.7 cells in a dose-dependent manner (Fig. 4I–K). Similarly, Western blot analysis showed that LPS markedly increased TNF-α expression, which was dose-dependently inhibited by SADS treatment (Fig. 4L and M). These findings suggest that the aqueous extract of SADS plays a critical role in modulating the inflammatory response by regulating the expression of key inflammatory cytokines, providing experimental evidence for its potential to attenuate the progression of RA.

### 3.4 SADS alleviated RA symptoms in IL-1RA^−/−^ mice

**Fig. 5:**
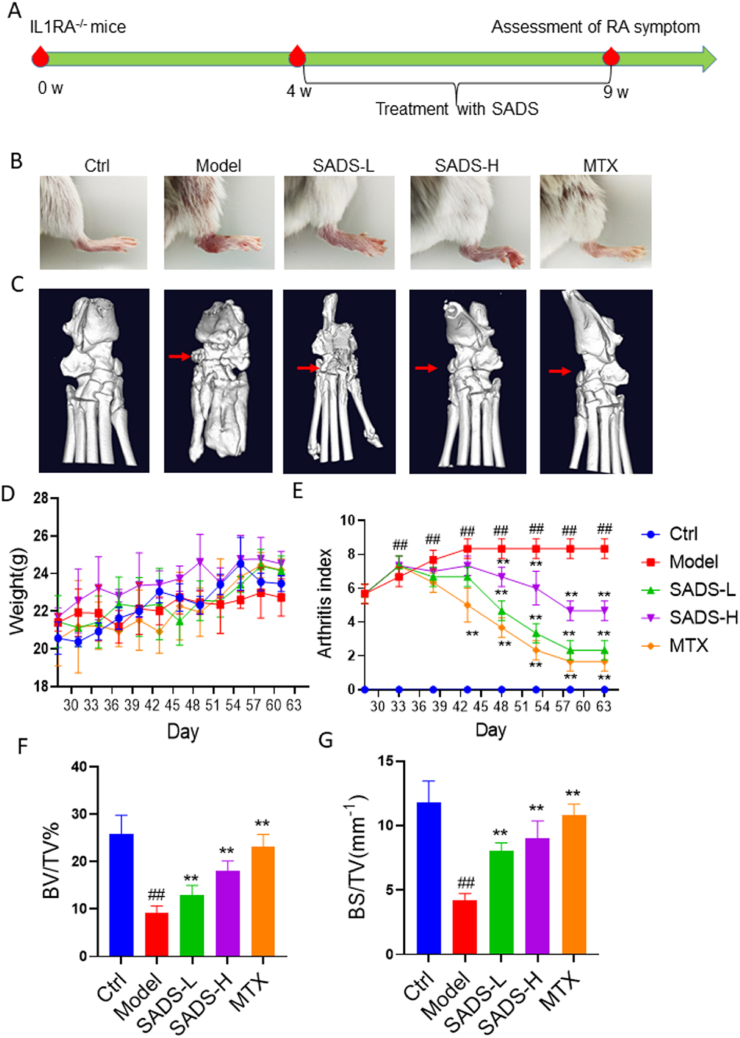
SADS ameliorated RA symptoms in IL-1RA deficient mice.(A) Animal experiment scheme.(B) Foot image in IL-1RA^−/−^ mice.(C) Micro-CT image of foot in IL-1RA^−/−^ mice.(D) Body weight change recorded every 3 days in IL-1RA^−/−^ mice from day 27. (E) Arthritis index recorded every 5 days in IL-1RA^−/−^ mice from day 27. (F and G) Ankle BV/TV% and BS/TV (mm^−1^) in IL-1RA^−/−^ mice. ##P < 0.01 versus Ctrl; ∗∗P < 0.01versus Model. n = 6 mice for each group.

The IL-1RA deficient mouse model, generated through genetic engineering, is commonly used to study autoimmune diseases such as RA [40]. By knocking out the IL-1RA gene, this model removes the inhibition of pro-inflammatory cytokine IL-1, thereby activating its receptor and triggering inflammation, which leads to the spontaneous development of arthritis in mice. In this study, IL-1RA deficient mice were employed to evaluate the therapeutic effects of SADS on RA, and the experimental design is outlined in Fig. 5A.

Treatment with SADS-L, SADS-H, and MTX significantly reduced foot swelling in IL-1RA deficient mice (Fig. 5B). Micro-CT analysis revealed severe bone deformities in the feet of the IL-1RA deficient mice; however, these deformities were notably improved in the SADS and MTX treatment groups (Fig. 5C). While SADS had no significant effect on the body weight of arthritic mice (Fig. 5D), it significantly improved the arthritis severity, as indicated by the arthritis score (Fig. 5E). Analysis of BV/TV% in the ankle joints using Analyze12.0 software showed a significant decrease in the model group due to joint expansion. In contrast, the BV/TV% in the SADS-treated groups increased progressively in a dose-dependent manner (Fig. 5F). The BS/TV analysis showed a similar trend, further supporting the positive effects of SADS on bone integrity (Fig. 5G).

### 3.5 Pathological evaluation of synovial tissue in IL-1RA^−/−^ mice

**Fig. 6:**
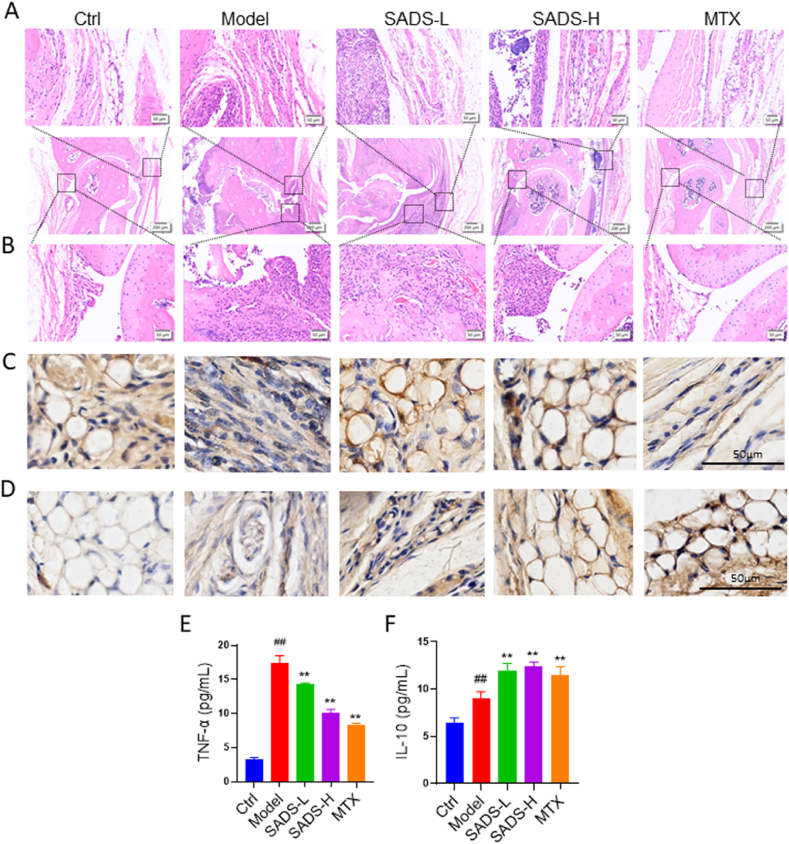
Pathological evaluation of IL-1RA^−/−^ mice synovial tissue. HE staining showed synovial hyperplasia (A) and inflammation (B). (C) IHC staining of TNF-α in synovial tissue. (D) IHC staining of IL-10 in synovial tissue.(E and F) Levels of TNF-α and IL-10 in the blood of IL1RA^−/^ -deficient mice. ##P < 0.01 versus Ctrl; ∗∗P < 0.01versus Model. n = 6 mice for each group. Control is wild-type mice, and the model is IL1RA^−/−^ mice.

Synovial tissue pathology was assessed using histological staining. As shown in Fig. 6A and B, the model group exhibited extensive synovial hyperplasia accompanied by inflammatory cell aggregation. Synovial hyperplasia was alleviated in the SADS-L and SADS-H groups, although inflammation was still evident. In contrast, synovial hyperplasia and inflammation were significantly suppressed in the MTX group. TNF-α staining revealed a marked increase in TNF-α expression in the synovial tissue of the model group. Compared with the model group, TNF-α levels were significantly reduced in the SADS-L and SADS-H groups, while the MTX group exhibited TNF-α expression levels close to normal (Fig. 6C). IL-10 staining showed that its expression was markedly increased in the SADS-L, SADS-H, and MTX groups but remained low in the control and model groups (Fig. 6D). In addition, serum TNF-α and IL-10 levels were evaluated in IL-1RA^−/−^ mice. Both SADS and MTX treatments significantly reduced TNF-α levels while markedly increasing IL-10 expression (Fig. 6E and F). These findings suggest that SADS may inhibit synovial hyperplasia by ameliorating synovial inflammation.

### 3.6 Pathological evaluation of ankle bone erosion in IL-1RA^−/−^ mice

**Fig. 7:**
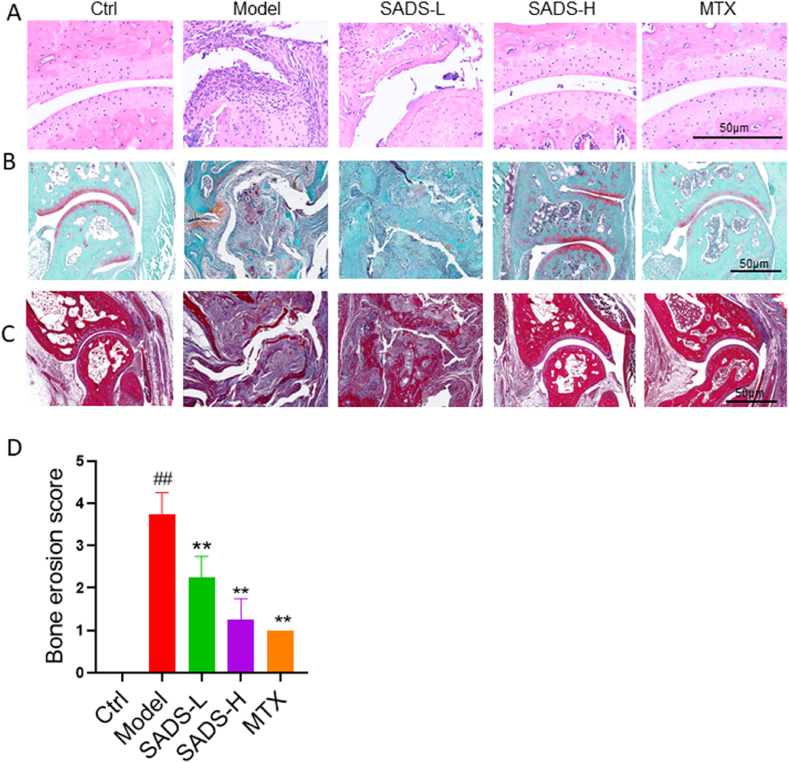
Pathological evaluation of ankle bone erosion in IL-1RA^−/−^ - mice.(A) HE staining of ankle bone tissue.(B) Saffron solid green staining of ankle bone tissue.(C) Masson staining of ankle bone tissue.(D) Ankle bone erosion score. ##P < 0.01 versus Ctrl; ∗∗P < 0.01versus Model. n = 6. Control is wild-type mice, and the model is IL1RA^−/−^ mice.

H&E staining results revealed severe bone defects in the model and SADS-L groups, while the ankle bones in the control, SADS-H, and MTX groups remained relatively intact, showing no signs of bone defects (Fig. 7A). Safranin fast green staining of the ankle joint showed intact cartilage tissue in the control, SADS-H, and MTX groups, as indicated by the presence of red-stained cartilage. In contrast, cartilage was markedly reduced in the model and SADS-L groups (Fig. 7B), suggesting significant cartilage erosion in these groups. Masson staining for new bone tissue revealed large areas of blue-stained new bone in the model and SADS-L groups. In contrast, the control, SADS-H, and MTX groups predominantly exhibited mature bone tissue, labeled with red dye (Fig. 7C). Bone erosion scores were significantly lower in the SADS-L, SADS-H, and MTX groups compared to the model group (Fig. 7D), indicating a marked improvement in ankle bone erosion in IL-1RA^−/−^ mice.

### 3.7 Non-targeted metabolomics analysis of anti-inflammatory active components in SADS-administered serum metabolites

**Fig. 8:**
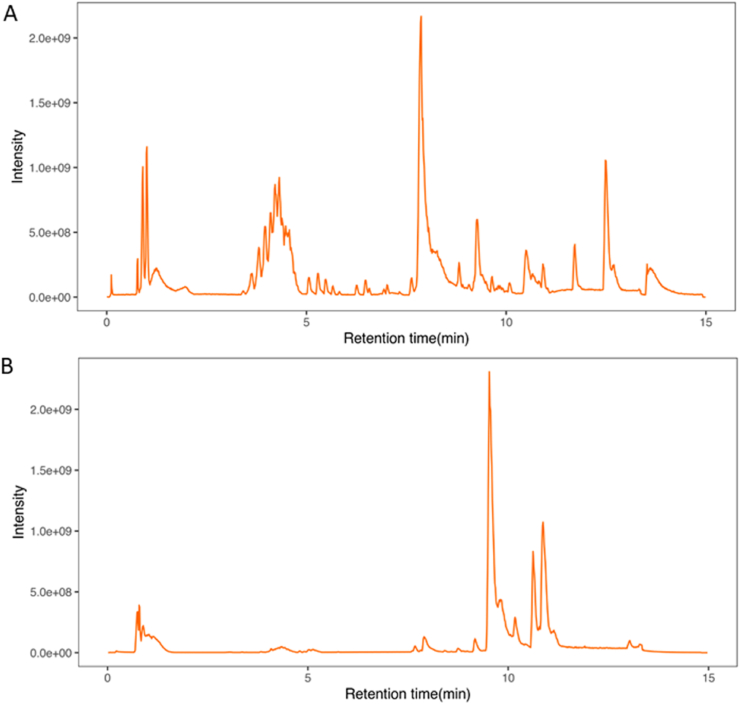
Total ion current chromatograms of SADS-administered serum in positive (A) and negative (B) ion mode.

**Fig. 9:**
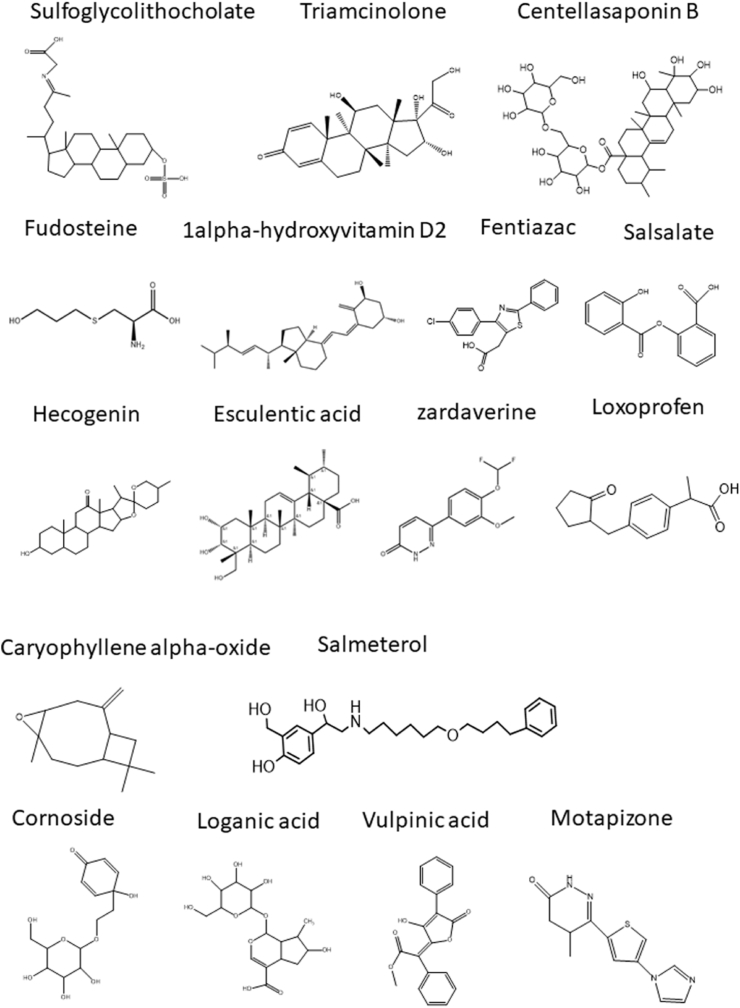
The structures of 17 Metabolites with anti-inflammatory effects in SADS-administered serum.

Non-targeted metabolomics analysis was conducted to investigate the anti-inflammatory components in the serum metabolites of SADS-treated mice. The presence of these active ingredients directly reflects the therapeutic effects of the drug [41]. UPLC-Q-TOF-MS was employed to detect the anti-inflammatory components in the serum of mice with spontaneous arthritis (Fig. 8). A total of 4945 metabolites were identified, of which 17 exhibited clear anti-inflammatory effects, with their structural formulas shown in Fig. 9. Among these, 13 anti-inflammatory compounds were common across all groups, and their levels were significantly higher in the SADS-H group compared to the model group (Table 1). Furthermore, four additional anti-inflammatory compounds Motapizone, Vulpinic acid, Loganic acid, and Cornoside were exclusively present in the SADS-L, SADS-H, and MTX groups (Table 2). The presence of these metabolites provides strong evidence that SADS can effectively benefit RA.

**Table 1:** Metabolites with anti-inflammatory effects in SADS-administered serum.

Metabolite	Ctrl	Model	SADS-L	SADS-H	MTX
Sulfoglycolithocholate	1.53	1.00	3.02	2.87	48.91
Triamcinolone	3.61	1.00	27.53	2.91	72.45
Centellasaponin B	2.98	1.00	9.47	1.00	20.43
Fudosteine	5.06	1.00	20.02	6.55	29.79
1alpha-hydroxyvitamin D2	6.65	1.00	1.08	4.36	18.39
Fentiazac	1.05	1.00	2.78	2.18	1.96
Hecogenin	2.28	1.00	3.37	1.84	2.28
Salmeterol	76.90	1.00	20.75	32.61	46.40
Caryophyllene alpha-oxide	1.10	1.00	0.88	1.83	0.38
Esculentic acid (Phytolacca)	13.20	1.00	4.40	7.44	3.75
Salsalate	7.72	1.00	1.11	4.05	1.64
Loxoprofen	0.87	1.00	0.60	1.03	0.51
zardaverine	3.47	1.00	12.13	1.28	5.29

Note: The relative abundance of all compounds was compared with the model group.

**Table 2:** Absent in the control and model groups, but emerging metabolites with anti-inflammatory effects in SADS-administered serum.

Metabolite	Ctrl	Model	SADS-L	SADS-H	MTX
Motapizone	0	0	2.14	1.00	4.22
Vulpinic acid	0	0	2.97	1.00	3.38
Loganic acid	0	0	204.74	1.00	1121.33
Cornoside	0	0	4.76	1.00	26.44

Note: The relative abundance of all compounds was compared with the SADS-H group.

### 3.8 Network pharmacology of SADS-administered serum metabolites

**Fig. 10:**
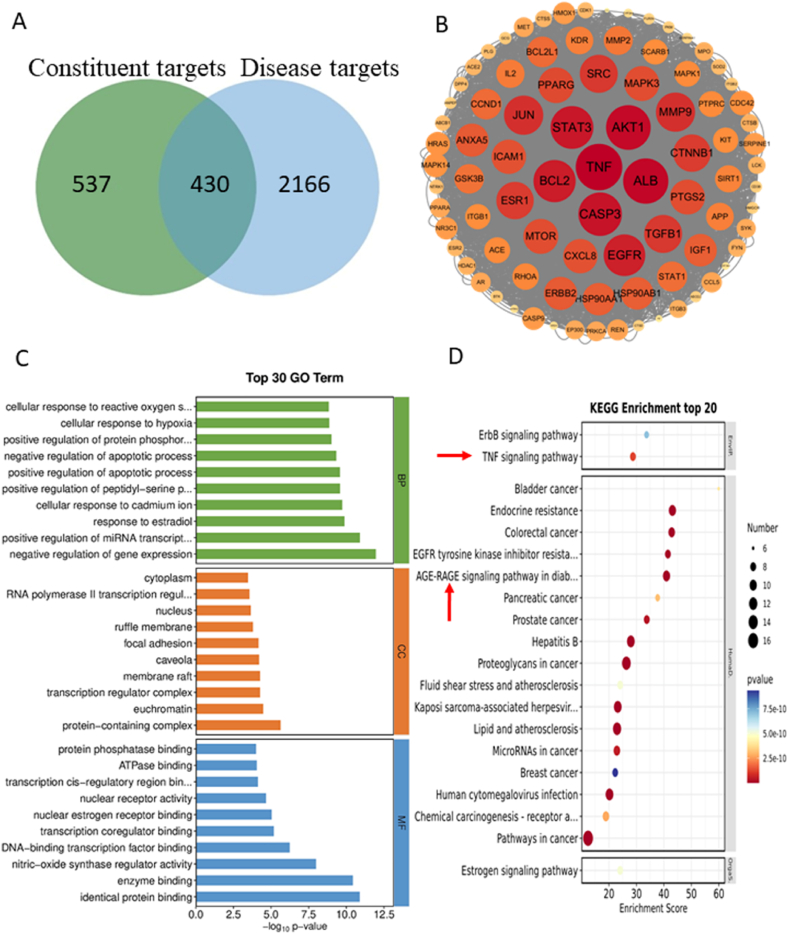
SADS-administered serum metabolites action targets and RA disease targets, as well as GO and KEGG pathway enrichment analysis. (A) Venn diagram of intersection between SADS-administered serum metabolites action targets and RA disease targets.(B) PPI network diagram of SADS-administered serum metabolites acting on RA disease targets.(C)GO enrichment analysis. (D)KEGG enrichment analysis.

The targets of the 17 identified anti-inflammatory active ingredients were first screened through public databases. Intersection with known “RA” disease targets yielded 430 potential therapeutic targets for SADS-administered serum metabolites in RA (Fig. 10A). Using the “Centiscape2.2″ tool, 87 targets with above-average “Degree”, “Closeness”, and “Betweenness” values were selected. These targets were ranked by Degree value using the “Network Analyzer” tool, resulting in a PPI network of 87 core targets (Fig. 10B). GO and KEGG pathway enrichment analyses were performed on these 87 targets to explore the potential mechanisms underlying the intervention of RA by SADS-administered serum metabolites (Fig. 10C and D). CC analysis highlighted the role of transcriptional regulatory complexes and protein-containing complexes, while MF analysis emphasized protein binding and nuclear receptor activity. Key RA-related pathways identified through KEGG analysis included TNF and RAGE signaling pathways. The action pathways of SADS-administered serum metabolites in RA closely resemble those of the active components in SADS, suggesting that TNF and RAGE may be key signaling pathways in the treatment of RA by SADS.

### 3.9 SADS prevented the abnormal growth of LPS-stimulated fibroblast-like synoviocytes through RAGE signaling pathway

**Fig. 11:**
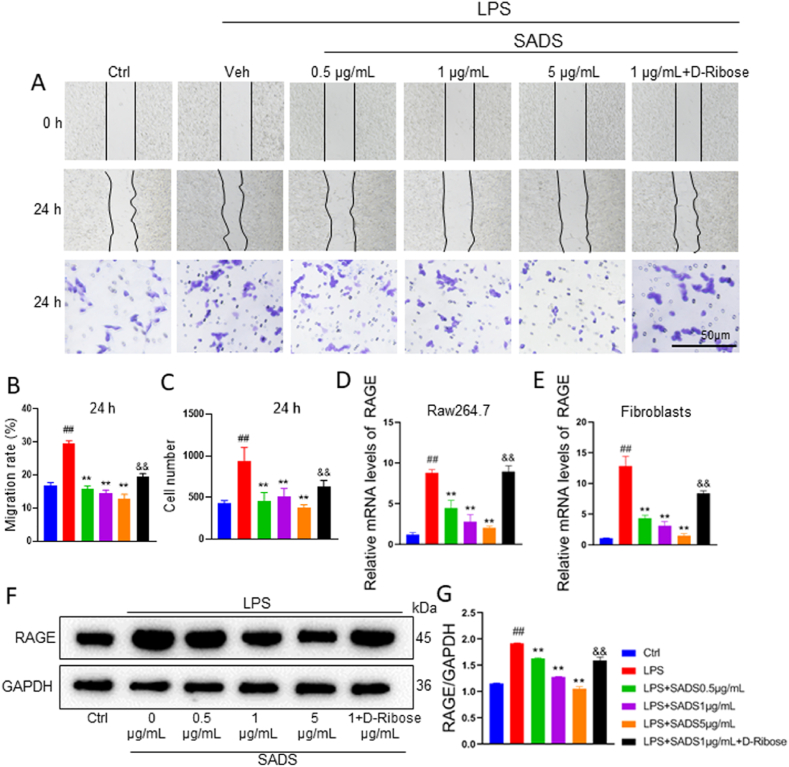
SADS prevented the abnormal growth characteristics of LPS-stimulated fibroblast-like synoviocytes through RAGE signaling pathway. (A) Scratch and transwell assays. (B) Fibroblast-like synoviocytes migration rate statistics. (C) Transwell experimental cell count. (D and E) The expression of RAGE in Raw264.7 and fibroblast-like synoviocytes was detected by RT-PCR. (F and G) RAGE protein expression level detection. ##P < 0.01 versus Ctrl; ∗∗P < 0.01versus LPS; &&P < 0.01versus SADS (1 μg/mL) n = 6.

Fibroblast-like synoviocytes play a pivotal role in the pathogenesis of RA by contributing to synovial hyperplasia, pannus formation, and joint destruction. Their aggressive, tumor-like behavior, particularly enhanced proliferation and migration, directly correlates with disease progression and joint damage [[42], [43], [44]]. The migration assays were included to evaluate whether SADS exerts inhibitory effects on one of the key pathological features of RA at the cellular levels. By assessing the migratory behavior of fibroblast-like synoviocytes *in vitro*, we aimed to investigate a potential mechanism by which SADS may reduce pannus formation and tissue invasion in the RA synovium. As such, the effects of SADS on LPS-induced fibroblast-like synoviocyte activation were evaluated using Scratch and Transwell assays. LPS stimulation significantly increased fibroblast-like synoviocyte migration, which was reduced by SADS intervention in a dose-dependent manner. However, d-Ribose (a RAGE agonist) restored the migration of fibroblast-like synoviocytes, counteracting the effects of SADS (Fig. 11A–C). RT-PCR analysis revealed a significant increase in RAGE mRNA expression in the LPS group, which was reversed by SADS in a dose-dependent manner. Notably, RAGE expression was markedly elevated in the d-Ribose group compared to the SADS-treated group (Fig. 11D and E). Protein-level analysis showed that LPS stimulation markedly upregulated RAGE expression, an effect significantly reversed by SADS treatment. Consistent with mRNA results, RAGE expression was elevated in the d-Ribose-treated group (Fig. 11F and G). These findings align with the network pharmacology analysis of SADS active ingredients and serum metabolites, confirming that the RAGE signaling pathway plays a key role in the therapeutic effects of SADS on RA.

## 4 Discussion

RA is a chronic autoimmune disease characterized by excessive secretion of inflammatory factors, including TNF-α and IL-1β, which drive fibroblast-like synoviocytes activation and subsequent cartilage destruction [45]. TCM has shown unique advantages in the treatment of RA due to its multi-target and holistic conditioning properties [46]. The core of TCM treatment of RA is syndrome differentiation [47]. According to the theory of TCM, RA belongs to the category of “Arthralgia syndrome”, which is caused by the invasion of wind, cold and damp pathogens and deficiency of liver-kidney qi and blood [48]. As a classical TCM, Wuweiganlu compound is able to exert anti-inflammatory and analgesic effects in RA by regulating the function of viscera, dredging meridians, removing rheumatism [29].

In the treatment of RA, over 130 TCM compounds have been reported, including SADS, Angelica sinensis, Licorice, and *Angelicae Pubescentis* Radix [49]. SADS is widely distributed in Asia, known as “Fang Feng” in China, “Bou-hu” in Japan, and “Bangpung” in Korea. In TCM, the root of SADS is generally used to treat wind dampness and cold diseases. SADS has been used in the treatment of RA because of its rich contents of anti-inflammatory active substances [15,16]. Although SADS water extract is a commonly used clinical drug preparation method, existing studies have shown that the content of active ingredients such as chromogen, coumarin and volatile oil in alcohol extract is higher [50,51], so the analysis and exploration of the content of these compounds in SADS alcohol extract, as well as their metabolites in blood and the therapeutic effect on RA will be our future focus of research.

Clinically, SADS was widely used in the treatment of inflammation, pain, and arthritis [52]. TCM can quickly relieve symptoms such as joint pain and swelling, improve joint function, and prevent disease progression by targeting mechanisms like the RAGE signaling axis [53].TCM formulations are rich in bioactive compounds such as alkaloids, polysaccharides, and glycosides, enabling systemic regulation. For instance, TCM treatments aim to modulate the immune system to improve local inflammatory microenvironments. The active ingredient of TCM, sinomenine, has been approved by the Chinese Food Supervision Administration for the treatment of RA due to its ability to inhibit the secretion of inflammatory factors [54]. Triptolide is one of the effective ingredients isolated from *Tripterygium wilfordii*, which has a good therapeutic effect on RA by reducing the levels of IL-1β and TNF-α [55]. Additionally, TCM improves gut microbiota composition, further supporting its systemic approach to RA treatment [56].

In this study, we identified 5536 compounds from SADS aqueous extracts and selected 19 active components. Non-targeted metabolomics analysis of SADS-administered serum identified 4945 compounds, including 17 with anti-inflammatory properties. Network pharmacology revealed that these active components exert therapeutic effects on RA primarily through TNF-α, RAGE, and IL-17 signaling pathways. Therefore, we suggest that SADS may treat RA through regulating the TNF-αand RAGE signaling pathways. WB analysis confirmed that SADS inhibits TNF-α and RAGE expression in fibroblast-like synoviocytes. Consistent with previous studies, key pathways such as TNF-α and RAGE were also identified in TCM compounds like Apigenin [53], Silibinin [57], Glabridin [58], EGCG [59], Coumarin [60]. Notably, SADS is rich in coumarin, further supporting its ability to mitigate RA progression by suppressing RAGE signaling. Interestingly, we found the metabolites of coumarin, Coumarin 6, Anisocoumarin H and 7,7' -dihydroxy-6,8′-bicoumarin, in blood metabolites. However, the blood metabolites of other compounds were rarely found, which may be due to the low content of chromogenone and coumarins in the water extract of SADS root, and the low amount of small molecular metabolites of these compounds absorbed into the blood after gastric acid decomposition. Therefore, future studies on the metabolites of alcohol extracts of SADS in blood may have unexpected results, which is also the focus of our future work.

The receptor for RAGE is a transmembrane protein in the immunoglobulin superfamily that contributes to the development of chronic diseases such as diabetes, osteoporosis, and RA [61]. Activation of RAGE by advanced glycation end products (AGEs) triggers inflammatory pathways. The abnormal expression of RAGE in the synovial tissues of RA and osteoarthritis patients underscores its role in inflammatory pathogenesis [62]. In this study, both SADS extracts and SADS-administered serum metabolites network pharmacological analysis results showed the important role of TNF-α and RAGE signaling pathway. Therefore, it is reasonable to believe that SADS exerts its therapeutic effect on RA through TNF-α and RAGE signaling pathways. Overall, this study highlights the pivotal role of TNF-α and RAGE signaling pathways in the therapeutic effects of SADS, as shown through SADS aqueous extract analysis and serum metabolite profiling.

In this study, UPLC-Q-TOF-MS technology and network pharmacology methods were used to clarify the mechanism of SADS on RA from cell, animal, and blood metabolite levels for the first time, and provide reference for the study of more than 130 traditional Chinese medicine compounds containing SADS in the treatment of RA. In the present study, network pharmacology analyses were only utilized to elucidate the molecular mechanism of SADS in the alleviation of RA. These results provide a valuable hypothesis-generating foundation for SADS treatment for RA, they require further experimental validation in future studies, such as RNA sequencing or proteomic profiling.

## 5 Conclusion

This study demonstrates the significant therapeutic efficacy of SADS in IL-1RA deficient mice with spontaneous arthritis. UPLC-Q-TOF-MS analysis identified 19 active components in SADS aqueous extracts and 17 anti-inflammatory components in serum metabolites. Network pharmacology revealed that these components target TNF-α and RAGE signaling pathways to exert anti-RA effects, which was further validated by WB analysis in fibroblast-like synoviocytes. These findings provide valuable insights into the molecular mechanisms underlying the therapeutic effects of SADS and establish a reference for further exploration of TCM formulations containing SADS in RA treatment, as well as theoretical support for its clinical applications.

## CRediT authorship contribution statement

**Anjing Xu:** Methodology, Formal analysis, Data curation, Conceptualization. **Yuanyuan Wen:** Project administration, Investigation. **Bao Hou:** Validation, Methodology, Funding acquisition, Formal analysis, Conceptualization. **Shijie Zhang:** Writing – original draft, Project administration, Formal analysis, Data curation. **Tsedien Nhamdriel:** Writing – review & editing. **Xiaoyue Ma:** Data curation. **Liyuan Cui:** Conceptualization. **Xuexue Zhu:** Funding acquisition. **Weiwei Cai:** Investigation, Funding acquisition, Formal analysis, Conceptualization. **Liying Qiu:** Writing – review & editing, Conceptualization. **Haijian Sun:** Writing – review & editing.

## Declaration of generative AI and AI-assisted technologies in scientific writing process

The authors used Kimi service to improve the readability and language of our manuscript. All authors have reviewed and edited the contents and will take full responsibility for the contents of the published article.

## Declaration of competing interest

All authors state that we have no known competing financial interests or personal relationships that might affect the work reported in this article.

## Data Availability

Data will be made available on request.
