# Supplementary figures and images for: Aqueous extract of Saposhnikovia divaricata root alleviates rheumatoid arthritis by acting on TNF-α and RAGE signaling pathways
Source: Biochem Biophys Rep. 2025 Jul 12;43:102153. doi: 10.1016/j.bbrep.2025.102153 (PMC12275060; doi:10.1016/j.bbrep.2025.102153)

$\beta$ -Actin-1

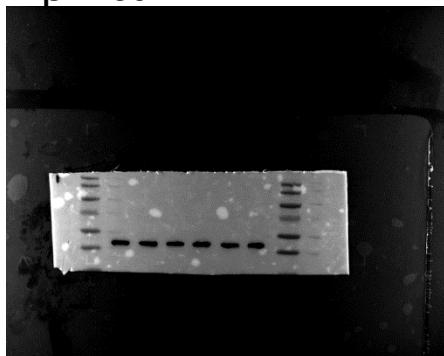

2

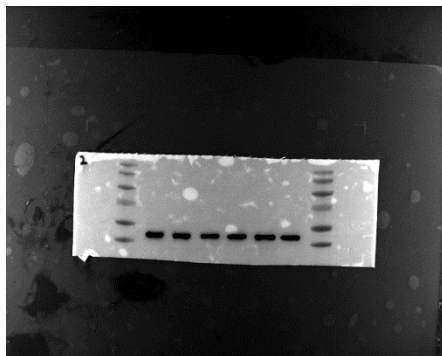

3

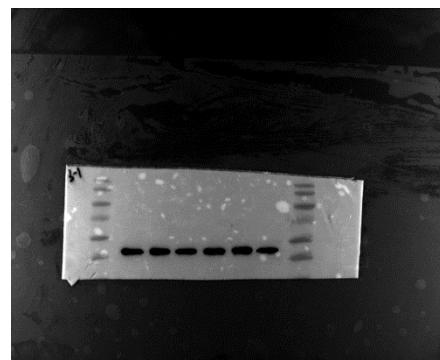

TNF- $\alpha$ -1

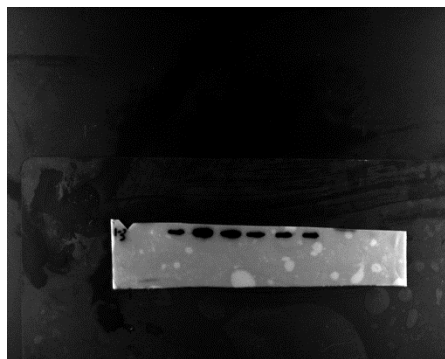

2

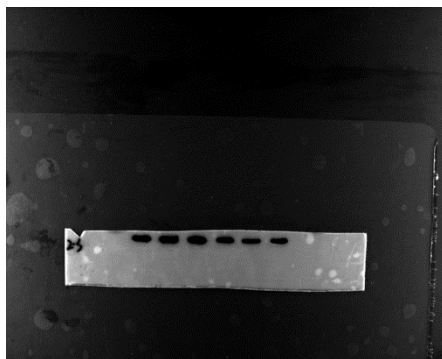

3

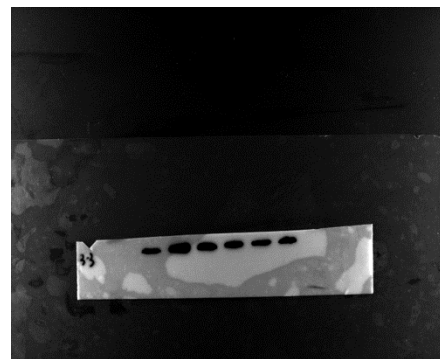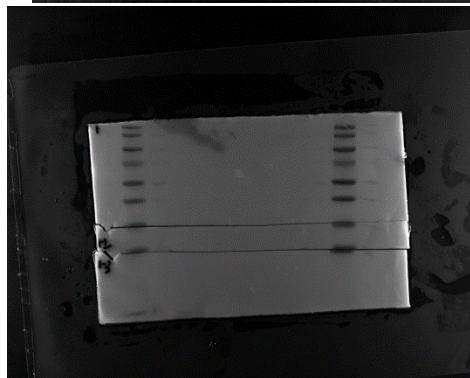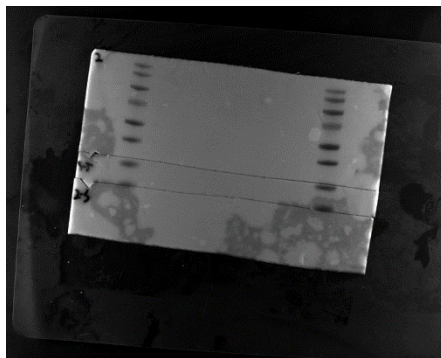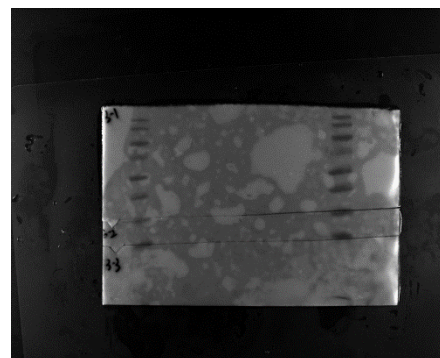

GAPDH-1

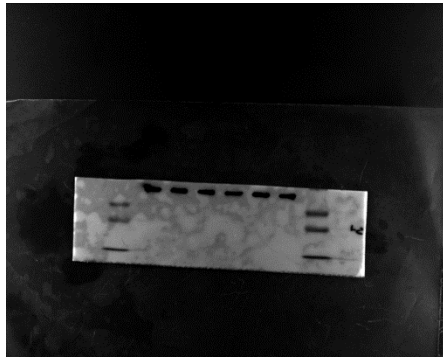

2

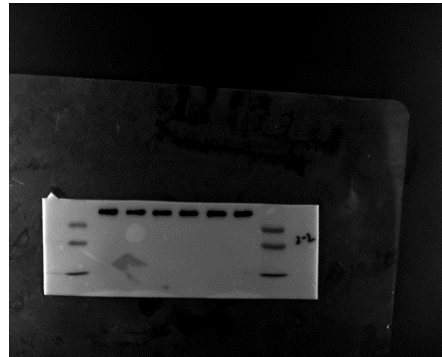

3

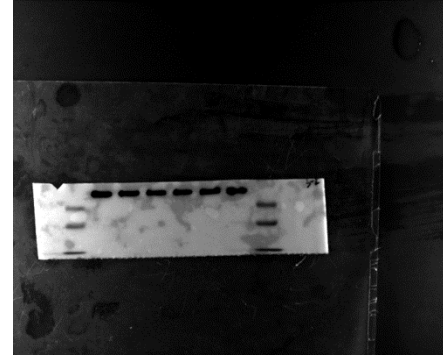

RAGE-1

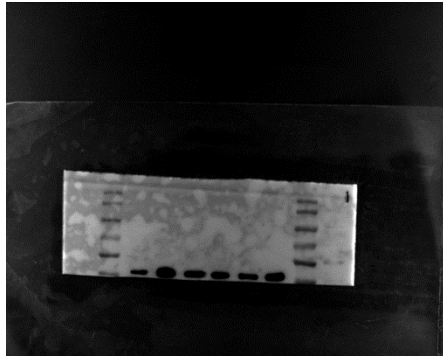

2

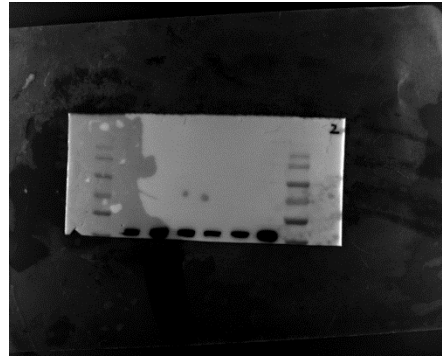

3

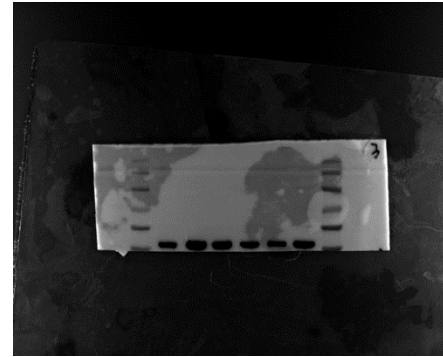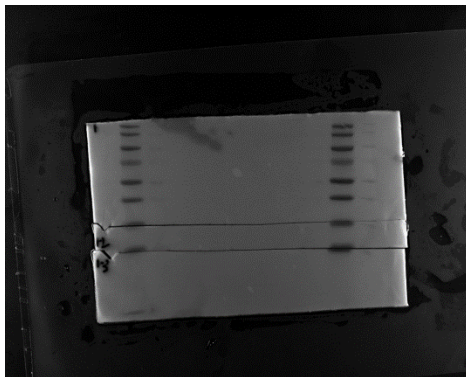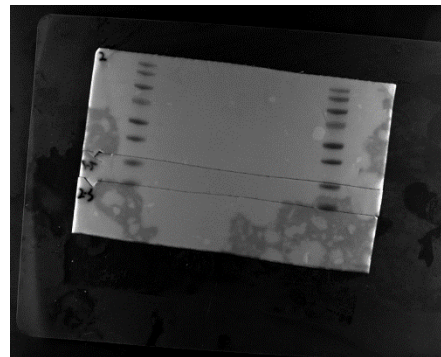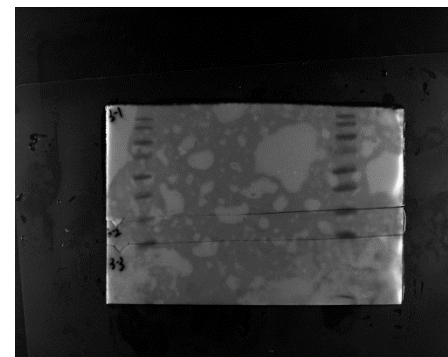

Supplement: Multimedia component 1 [file mmc1.pdf]
